# Retinal Angiomatous Proliferation: Multimodal Imaging Characteristics and Follow-up with Eye-Tracked Spectral Domain Optical Coherence Tomography of Precursor Lesions

**DOI:** 10.4274/tjo.03780

**Published:** 2018-04-25

**Authors:** Zafer Öztaş, Jale Menteş

**Affiliations:** 1 Ege University Faculty of Medicine, Department of Ophthalmology, İzmir, Turkey

**Keywords:** Retinal angiomatous proliferation, spectral-domain optical coherence tomography, type 3 neovascularization

## Abstract

**Objectives:**

To present the multimodal imaging characteristics of precursor retinal angiomatous proliferation (RAP) lesions and follow-up results with eye-tracked spectral-domain optical coherence tomography (SD-OCT).

**Materials and Methods:**

Six eyes of 6 patients aged 77.5±5.9 years diagnosed with precursor RAP lesion were included in this prospective observational case series. Best corrected visual acuity (BCVA) measurement and complete ophthalmologic examination were performed for all subjects, as well as fundus photography (FP), fundus autofluorescence (FAF), SD-OCT, fluorescein angiography (FA), indocyanine green angiography (ICGA), optical coherence tomography angiography (OCTA), and their long-term follow-up results are presented.

**Results:**

The mean BCVA was 0.8±0.16 (0.6-1) Snellen and the mean follow-up was 26.3±14.8 months. Images of the precursor RAP lesions demonstrated no specific findings on FP and FAF, showed focal hypofluorescent foci with no leakage on FA and IGA, and appeared as extrafoveal small, round, well-defined, hyperreflective foci typically located in the outer retinal layers on SD-OCT B-scans with high sampling density. OCTA demonstrated the precursor lesions as the deep capillary plexus abnormalities in 3 eyes. Two eyes progressed to stage 1 RAP during the follow-up period.

**Conclusion:**

This study defined the diagnostic characteristics and clinical course of precursor RAP lesions. Our findings highlight the importance of B-scans with high sampling density for the diagnosis of precursor lesions and using eye-tracking mode SD-OCT during follow-up.

## Introduction

The term retinal angiomatous proliferation (RAP), first used by Yannuzzi in 2001, describes a specific clinical form of age-related macular degeneration (AMD) characterized by neovascularization (nv) originating from the outer layers of the retina.^1^ It is known that the initial stage (precursor lesion) of RAP, also called type 3 nv, involves the development of an angioma (neovascular complex) originating from the deep capillary network (DCN) of the retina. The disease progresses to advanced stages (stages 1, 2, and 3) as this highly vasogenetic angioma proliferates and progresses toward the pigment epithelium and choroidal layers, leading to vision loss.^1,2,3,4,5^

The rapid clinical course and typical lack of response to anti-VEGF (vascular endothelial growth factor) therapy in patients with advanced RAP make the early detection and monitoring of precursor lesions of great importance.^2,3,4,5^

In this article, we describe the multimodal imaging features of precursor RAP lesions, including optical coherence tomography angiography (OCTA), and present the results of long-term follow-up with B-scan spectral domain optical coherence tomography (SD-OCT) using high sampling density and eye-tracking mode.

## Materials and Methods

This prospective, observational clinical series included a total of 6 eyes of 6 patients (4 females and 2 males; mean age 77.5±5.9 [71-87] years) diagnosed with bilateral AMD. All of the patients were receiving anti-VEGF therapy for nvAMD in the other eye when precursor (early-stage) RAP lesions were incidentally detected on B-scan SD-OCT imaging. All patients underwent best corrected visual acuity (BCVA) measurement and full ophthalmologic examination, as well as fundus photography (FP), fundus autofluorescence (FAF), SD-OCT, fluorescein angiography (FA), indocyanine green angiography (ICGA), and OCTA examinations, and diagnostic features were determined. The patients’ symptomatic eyes were already being followed, and their asymptomatic fellow eyes were also observationally examined in detail. Informed consent was obtained from all patients. 

Precursor RAP lesions were diagnosed based on the appearance of hyperreflective lesions in the outer layers of the retina that originated from the DCN and were characteristically round and well-defined, and typically caused shadowing at the level of the retinal pigment epithelium (RPE) on B-scan SD-OCT. These lesions had not yet caused any intraretinal and/or subretinal fluid accumulation or increase in retinal thickness. Their course and progression over time were monitored at intervals of one or two months using B-scan OCT in eye-tracking mode with high sampling density. 

BCVA measurements, anterior and posterior segment examinations, and SD-OCT scanning were repeated at each visit. In addition to FP, FAF, FA, ICGA, and high-density eye-tracked B-scan SD-OCT images acquired using a Heidelberg Spectralis HRA-OCT (Heidelberg Engineering, Heidelberg, Germany), a Topcon OCT-2000 (Topcon, Tokyo, Japan) device was also used to record color photographs, en face (C scan), and three-dimensional (3D) images. OCTA images were acquired in a 3x3 mm area with 11-µm B-scan intervals using the Heidelberg Spectralis HRA + OCT OCT 2 module (Heidelberg Engineering, Heidelberg, Germany).

## Results

All of the eyes were asymptomatic and had BCVA of 0.8±0.16 (0.6-1) Snellen at time of diagnosis. Mean follow-up time was 26.3±14.8 months. The lesions were accompanied by drusenoid pigment epithelial detachment (PED) in 4 eyes and by drusen in 2 eyes. Two of the eyes exhibited multiple hyperreflective lesions.

**Figure 1:**
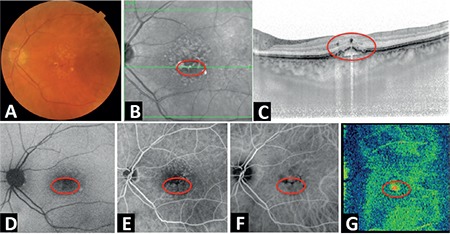
The multimodal imaging characteristics of a precursor retinal angiomatous proliferation lesion (in red circle) (patient 1). A) Drusen and pigment deposits on color fundus photography; B) small, dark areas on infrared fundus photography; C) precursor lesion and typical back shadowing are seen on B-scan spectral-domain optical coherence tomography; D) small hypoautofluorescent area on fundus autofluorescence; E) no leakage in fluorescein angiography; F) no leakage in indocyanine green angiography, G) extrafoveal small, round, well-defined, hyperreflective foci in the outer retinal layers on C-scan (en face) spectral-domain optical coherence tomography

**Figure 2:**
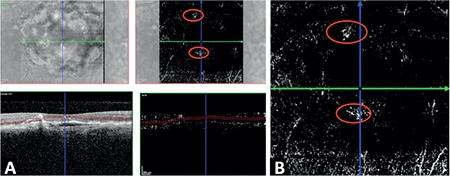
In optical coherence tomography angiography (OCTA) images of patient 1, the precursor retinal angiomatous proliferation lesion (in red circle) appeared as a hyperreflective, small, round, well-defined microvascular tuft at the outer capillary plexus level: A) OCTA image, B) magnified image of the OCTA section

The precursor RAP lesions had no specific findings in clinical examination or color FP; however, they appeared as small, darkly colored areas on infrared FP and FAF, and as focal hypofluorescent foci with no leakage on FA and ICGA (Figure 1). On SD-OCT, they appeared as small, round, well-defined extrafoveal hyperreflective foci located in the outer layers of the retina (between the outer plexiform layer and outer nuclear layer) in both B-scan and in en-face and 3D images. All of the lesions were noted to cause typical back shadowing at the RPE level on B-scan OCT (Figure 1). Precursor RAP lesions could only be viewed with OCTA examination in 3 eyes (50%), and they were usually observed as a hyperreflective, small, round, well-defined microvascular tuft at the outer capillary plexus level (Figure 2). 

**Figure 3:**
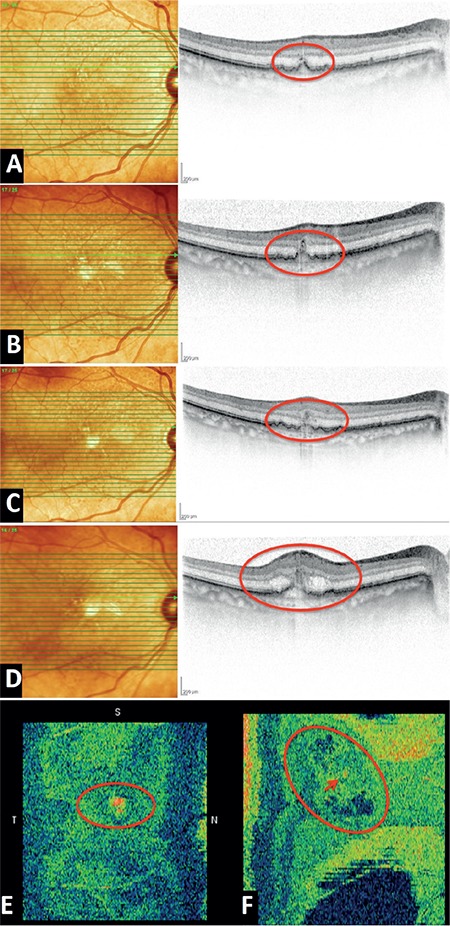
Progression of precursor lesion to stage 1 retinal angiomatous proliferation (RAP) in patient 2: A) The precursor RAP lesion (in red circle) with typical back shadowing in the right eye; B) the same section 6 months later shows the lesion changing form and approaching the retinal pigment epithelium; C) another image obtained 1 month later shows increased retinal thickness; D) intraretinal cystoid fluid accumulation is seen in the retina 2 months later. E) En face SD-OCT image of the precursor lesion; F) progression to stage 1 RAP and cystoid spaces formed around the precursor lesion (red arrow)

During follow-up, the precursor RAP lesions remained stable in 4 eyes and became active in the other 2 eyes after an average of 21 months (at 12 and 30 months). The activated angiomas showed slight growth on OCT, enlarging and shifting slightly downward toward the RPE layer and/or the drusenoid PED (Figure 3). One to two months after these changes, these two patients exhibited a sudden, small decrease in BCVA (6-7/10 Snellen), together with OCT findings of increased retinal thickness and intraretinal cystoid fluid formation, indicating progression to stage 1 RAP.

## Discussion

In this article, we present the multimodal imaging features of 6 eyes diagnosed with precursor RAP lesions as well as their high-density eye-tracked B-scan OCT findings from a mean follow-up period of 26.3±14.8 (6-42) months. RAP precursor lesions, or intraretinal neovascular complexes, are quite small, and our study emphasizes the importance of using high sample density B-scan SD-OCT imaging passing directly over the lesion as well as using eye-tracking mode, which yields reproducible images, in the diagnosis of precursor RAP lesions. 

Precursor RAP lesions do not show leakage on FA or ICGA and have typical features on SD-OCT. Recognizing these lesions in the earliest asymptomatic stage, before the appearance of intraretinal cystoid fluid and serous PED, is important because advanced disease is very aggressive and often resistant to anti-VEGF therapy.

Su et al.^5^ reclassified type 3 nv lesions according to SD-OCT findings. Although isolated punctate hyperreflective foci on the outer retinal layers were described as “precursor” lesions, the researchers acknowledged that hyperreflective foci may also appear in other macular diseases and that they alone do not constitute a specific sign of type 3 nv. They identified growth of precursor lesions, downward shift toward the retinal layers, and outer plexiform layer disruption as specific symptoms of activation, whereas they defined the presence of a larger precursor lesion accompanied by cystoid macular edema but without disruption of the outer retinal layers as stage 1 RAP. They reported that there may be a long period of time between the appearance of a precursor lesion and stage 1, with a mean interval of 3.6±3.3 months for the patients in their series. 

The results of our study demonstrate that these very small hyperreflective lesions in the outer retinal layers, which we determined as being earliest stage RAP lesions and which were identified by Su et al.^5^as precursor lesions, can easily be identified with high-density SD-OCT. During follow-up in our patients, activation and progression to stage 1 were detected after an average of 21 months following initial diagnosis in 2 (33.2%) eyes. By diagnosing progression in the early stages, we were able to initiate treatment before permanent changes occured in the outer retinal layers.

Querques et al.^2^ described the multimodal imaging features of their clinical series including type 3 nv patients with precursor lesions. All precursor lesions in their study were located over drusen or drusenoid PED, and they reported a 32% rate of progression from precursor lesion to stage 1 at a mean interval of 19.6±9.5 months after diagnosis. As we also observed in the patients in the present series, they noted that precursor lesions were hypofluorescent on FA and ICGA during their inactive period, but became hyperfluorescent or exhibited leakage after the appearance of signs of activation and after progression to  stage 1.

Tan et al.^6^ described the OCTA findings of active or inactive early type 3 nv patients. Their study did not include precursor lesions, and imaging rate was reported as 85%.

## Conclusion

Our study using multimodal imaging to evaluate the diagnostic features and clinical course of precursor RAP lesions emphasizes the importance of high-density B-scan SD-OCT imaging for diagnosis and using eye-tracking mode to better detect possible activation during follow-up.
